# Dietary Sulfur Amino Acid Restriction and the Integrated Stress Response: Mechanistic Insights

**DOI:** 10.3390/nu11061349

**Published:** 2019-06-15

**Authors:** William O. Jonsson, Nicholas S. Margolies, Tracy G. Anthony

**Affiliations:** Department of Nutritional Sciences, Rutgers University, New Brunswick, NJ 08901, USA; william.jonsson@rutgers.edu (W.O.J.); margoliesnick@gmail.com (N.S.M.)

**Keywords:** fibroblast growth factor 21, cystathionine gamma lyase, healthspan, liver, dietary restriction, amino acid stress

## Abstract

Dietary sulfur amino acid restriction, also referred to as methionine restriction, increases food intake and energy expenditure and alters body composition in rodents, resulting in improved metabolic health and a longer lifespan. Among the known nutrient-responsive signaling pathways, the evolutionary conserved integrated stress response (ISR) is a lesser-understood candidate in mediating the hormetic effects of dietary sulfur amino acid restriction (SAAR). A key feature of the ISR is the concept that a family of protein kinases phosphorylates eukaryotic initiation factor 2 (eIF2), dampening general protein synthesis to conserve cellular resources. This slowed translation simultaneously allows for preferential translation of genes with special sequence features in the 5′ leader. Among this class of mRNAs is activating transcription factor 4 (ATF4), an orchestrator of transcriptional control during nutrient stress. Several ATF4 gene targets help execute key processes affected by SAAR such as lipid metabolism, the transsulfuration pathway, and antioxidant defenses. Exploration of the canonical ISR demonstrates that eIF2 phosphorylation is not necessary for ATF4-driven changes in the transcriptome during SAAR. Additional research is needed to clarify the regulation of ATF4 and its gene targets during SAAR.

## 1. Sulfur Amino Acid Restriction as a Dietary Strategy to Promote Leanness and Longevity

Understanding how dietary protein quantity versus quality impacts growth and health has been studied and debated for over 100 years [1,2]. The idea that reduction of individual amino acids can slow growth and aging was identified several decades ago [3]. In 1993, Orentreich et al. published a seminal report revealing the effects of dietary methionine restriction on lifespan extension in male Fischer 344 (F344) rats [4]. By restricting the methionine content from 0.86 to 0.17 g per 100 g diet (~80% reduction) throughout the adult lifespan of the animals, the authors observed an approximate 40% extension in average lifespan compared to unrestricted rats. Coupled with the observation that methionine restricted animals maintained a lower body weight while increasing their relative food intake, this report concluded that dietary methionine restriction eliminates growth while increasing lifespan. Since this original publication, dietary methionine restriction has gained interest as a research model to aid in the prevention and treatment of obesity-related metabolic diseases.

Subsequent investigations in rodent models indicate that the range of dietary methionine restriction which elicits leanness without protein wasting and food aversion is 0.12 to 0.25 g per 100 g diet, as compared to the 0.43 to 0.86 g per 100 g in complete rodent diets [5,6]. However, most studies to date utilize methionine levels ranging between 0.12 to 0.17 g per 100 g diet with 0.17 g per 100 g diet the most well-studied restriction level. It is also important to note that the majority of methionine restriction diets are based on a zero-cysteine dietary background. Work by several labs have emphasized the importance of coupling methionine restriction with total dietary depletion of cysteine, the other major dietary sulfur amino acid [6,7,8,9]. Indeed, metabolic and body weight changes are completely reversible with the addition of 0.2 g cysteine per 100 g diet to a methionine restricted diet [10]. Consequently, methionine restriction may be more accurately described as sulfur amino acid (SAA) restriction (SAAR).

Much of the recent interest paid to SAAR stems from the observation that rodents subjected to the restricted diet display increased healthspan. Health improvements induced by SAAR include attenuation of body weight and body fat gain. Investigation into the altered body composition seen in rodents fed SAAR points to increases in energy expenditure as well as heat increment of feeding [11]. Dietary SAAR also reduces fat deposition, both overall and in inguinal, epididymal, and retroperitoneal fat pads, and simultaneously induces browning of white adipose tissue [11,12]. This reduction in fat deposition is the combined result of increased expression of fatty acid oxidation genes in adipose tissue and decreased expression of lipogenic genes in liver and white adipose, contributing to a shift towards fatty acid oxidation on a whole-body level [11,13]. Additionally, triglyceride content in serum and liver is decreased [12,13]. Notably, SAAR ameliorates liver steatosis associated with diet-induced obesity and prevents progression of hepatic steatosis in *ob*/*ob* mice [13,14]. Furthermore, SAAR prevents type 2 diabetes in New Zealand Obese mice [15].

The reduced overall adiposity in SAA restricted rodents corresponds with reductions in fasting concentrations of insulin, glucose, thyroxine, insulin-like growth factor-1 and leptin, and increases in serum adiponectin [5,11,14,16,17]. Part of the mechanism behind the improved fasting insulin is dependent on SAAR-mediated increased sensitivity to insulin-dependent Akt phosphorylation in the liver [17]. In addition, obese mice subjected to dietary SAAR display increased plasma membrane localization of the GLUT4 glucose transporter and glycogen synthesis in gastrocnemius muscle, potentially contributing to improved insulin sensitivity in conjunction with SAAR [18]. 

Other systemic effects of SAAR include delayed cataract development, downregulation of arrhythmogenic, hypertrophic, and cardiomyopathy signaling pathways in the heart, and attenuated cardiac response to beta adrenergic stimulation [19]. On the other hand, dietary SAAR may contribute to reduced bone mass and altered intrinsic and extrinsic bone strength. Notably, recent findings suggest that male mice subjected to SAAR display decreased bone tissue density in both trabecular and cortical bone, simultaneous with an observed induction in fat accumulation in bone marrow [20]. As bone mass and quality are important predictors of health with advancing age, this topic remains to be further explored in greater detail [14,21].

At a glance, SAAR appears to recapitulate many of the beneficial effects attributed to caloric restriction; however, it is worth noting that SAAR elicits a transcriptional response in liver that partly differs from caloric restriction [22]. Furthermore, the specific transcriptional response to insufficiency of different single amino acids shows that deprivation or restriction of methionine elicits a hepatic response that is divergent from restriction of the other essential amino acids [22,23,24]. Taken together, the current literature supports a view in which SAAR, within a limited range of intakes, improves metabolic health by uniquely altering target tissues. 

While the currently available literature shows robust physiological improvements with SAAR in rodent models, the underlying mechanisms are only partly understood and are subject to ongoing research. Among the known nutrient-responsive signaling pathways, the evolutionary conserved integrated stress response (ISR) is a lesser-understood candidate in mediating leanness and/or longevity by SAAR. Therefore, the purpose of this review is to compile and delineate the current understanding of the involvement of the ISR in mediating pro-adaptive responses to SAAR in mammals. 

## 2. The Integrated Stress Response and Detection of Amino Acid Insufficiency

Throughout evolution, all living organisms have encountered periods of nutrient scarcity. In order to ensure survival during such periods, intricate and overlapping cellular processes have evolved to promote resilience and metabolic homeostasis. Many of these signaling networks are evolutionary well-conserved. Among these networks, the ISR is identified in all eukaryotic organisms as a means to allow for conservation of resources to adapt to environmental stress, ultimately improving survivability [25]. 

A key feature of the classical or canonical ISR is the concept that a variety of cellular stresses are sensed by a family of protein kinases which together function as stress response regulators. These ISR regulators are: Protein Kinase R (PKR), which is stimulated by viral double stranded RNA; PKR-like endoplasmic reticulum kinase (PERK), which is activated by ER stress; heme regulated inhibitor (HRI), which modulates globin synthesis in response to heme deprivation; and general control nonderepressible 2 (GCN2), which senses amino acid insufficiency and cellular damage by UV light. 

Activation of these ISR regulators converge at the point of phosphorylation of the GTPase activating protein, eukaryotic initiation factor 2 (eIF2) at serine 51 of its α subunit. This covalent modification converts eIF2 into a competitive inhibitor of its guanine nucleotide exchange factor, eIF2B [26,27]. Inhibition of eIF2B then slows the rate at which eIF2 can be re-loaded with GTP. Ultimately, reduced rates of GTP-GDP exchange on eIF2, an essential step in mRNA translation re-initiation, alters gene-specific translation. 

**Figure 1:**
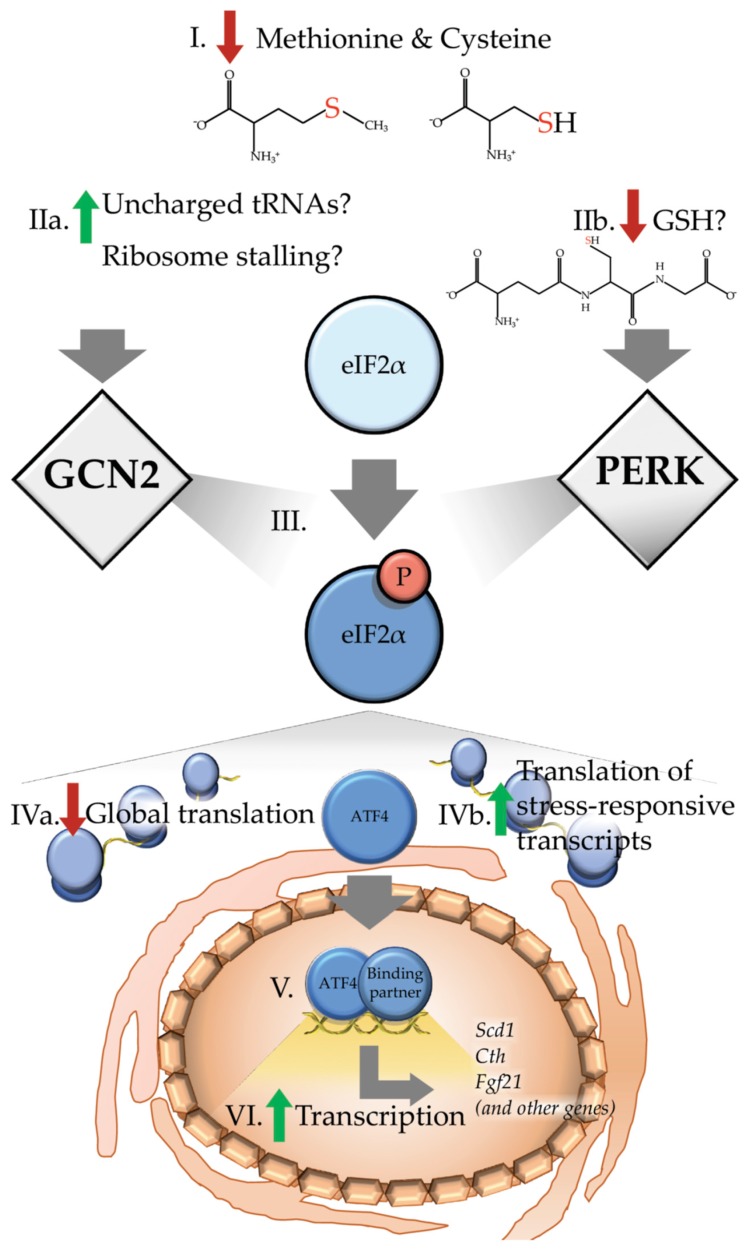
Dietary sulfur amino acid restriction results in activation of the integrated stress response. (I) Upon reduced levels of methionine and cysteine, a canonical integrated stress response (ISR) would predict or hypothesize that (IIa) levels of the uncharged cognate tRNAs may accumulate at or near the ribosome. Alternatively, other recent reports suggest increased ribosome stalling as a potential means of activating the eukaryotic initiation factor 2 (eIF2) kinase general control nonderepressible 2 (GCN2). In addition to activation of GCN2, dietary sulfur amino acid restriction (IIb) reduces levels of intracellular glutathione (GSH), which may in turn activate protein kinase R (PKR)-like endoplasmic reticulum kinase (PERK). Collectively, current evidence suggests that both of these paths results in (III) phosphorylation of the αsubunit of eIF2. Phosphorylation of eIF2 (IVa) decreases global translation (IVb) which increases preferential translation of transcripts containing upstream open reading frames such as the basic leucine zipper (bZIP) transcription factor *Atf4*. (V) Upon being translated, activating transcription factor 4 (ATF4) enters the nucleus and interacts with binding partners to (VI) induce transcription of target genes, including stearoyl-Coenzyme A desaturase 1 (*Scd1*), cystathionine gamma-lyase (*Cth*) and fibroblast growth factor 21 (*Fgf21*), as well as many other genes.

As one of the branches of the ISR, early detection of amino acid insufficiency by GCN2 functions to delay catastrophic depletion of the intracellular amino acid pool by reducing the bulk client load for protein synthesis (Figure 1). In brief, as cytosolic levels of specific amino acids decrease, aminoacylation levels of the cognate tRNAs also decline. These deacylated or ‘uncharged’ tRNAs bind GCN2 and activate the kinase through dimerization and autophosphorylation [28,29,30]. In addition, emerging evidence points towards sensing of ribosomal stalling as a potent activator of GCN2, plausibly mediated by the heteropentameric protein complex P-stalk [31,32]. 

While amino acid deprivation on the one hand triggers rapid reduction in global translation, it on the other hand leads to targeted increases in the translation of certain mRNAs which contain one or more upstream open reading frames (uORFs) in their 5′ leader or untranslated region (UTR) [33,34,35]. Within this subset of preferentially-translated mRNAs, the basic leucine zipper (bZIP) transcription factor, activating transcription factor 4 (ATF4), is best characterized much due to its central role in responding to amino acid insufficiency [33,36]. The *Atf4* transcript contains two uORFs which together allows for precise control of its translation: a 5′-proximal uORF1 that facilitates downstream ribosome re-initiation and a slightly longer uORF2 that is overlapping and out-of-frame with the *Atf4* coding sequence (CDS) [37]. Under well-nourished conditions, the structure of the *Atf4* transcript allows translation initiation at both uORFs, thus repressing synthesis of the CDS. In contrast, under conditions of cellular amino acid insufficiency there is reduced translation re-initiation efficiency and so scanning ribosomes bypass uORF2 in the *Atf4* transcript and instead re-initiate translation at the CDS [37]. 

Upon its translation, ATF4 translocates to the nucleus where it can form homodimers or heterodimers with other bZIP transcription factors and bind promoter sequence motifs referred to as CAAT enhancer binding proteinATF response elements (CAREs) or amino acid response elements (AAREs) [38]. Such response elements are contained within many genes encoding amino acid transporters and metabolic regulators, antioxidant defenses, cell cycle control, and other homeostatic processes, including negative feedback control, all collectively contributing to regain homeostatic conditions cellularly and ultimately on the organismal level following physiological stress [27,39,40,41]. 

In sum, the canonical ISR functions in the detection of amino acid insufficiency and contributes important information in decisions regarding cell fate. Adaptable stress such as that elicited by SAAR may be cytoprotective by promoting hormesis *via* the ISR. As such, the interplay between SAAR and the ISR demands more attention and thus has been subject to several recent investigations, summarized below. 

## 3. Restriction of Sulfur Amino Acids Reduces Protein Synthesis Independent of GCN2

In several growth restricted rodent models with long life, reduced protein synthesis is a shared response [42,43]. Following detection of reduced levels of intracellular amino acids by GCN2, global translation initiation is reduced to allow for conservation of resources and ultimately maintenance of protein homeostasis. Therefore, the reduced availability of essential sulfur amino acids may reduce protein synthesis. To that end, a number of reports have investigated the ability of SAAR to alter protein synthesis rates [10,44,45]. One study found that in the liver and skeletal muscle of mice fed SAAR for five weeks, protein synthesis rates are reduced in mixed and cytosolic protein fractions but not in the mitochondrial fraction [44]. The maintenance of mitochondrial protein synthesis is supportive of the notion that improved mitochondrial proteostasis supports longer lifespan of rodents [43]. In contrast, another study showed that SAA restricted male rats display reduced mixed fractional synthesis rates in liver but not in skeletal muscle nor in hippocampus [45]. Tissue-specific differences between studies may be due to the level of SAA restriction, a concept that requires further investigation.

While SAAR is shown to reduce rates of protein synthesis in tissues *in vivo*, how this dietary change is detected is less clear. Interestingly, one study found that the global loss of *Gcn2* did not prevent reductions in protein synthesis rates in both liver and skeletal muscle. Instead, *Gcn2* deleted mice fed an SAA restricted diet retained more body fat as compared to intact mice. This finding differs from that reported in *Gcn2* deleted mice fed a leucine-devoid diet which showed sustained liver protein synthesis at the expense of muscle mass and greater body weight loss as compared to intact mice [46]. These differences may be a function of the timing of the measurement, choice of amino acid deprivation or age of the mice, or the combination of these factors. 

Of interest, several reports collectively show that while GCN2 may be activated by SAAR, it is not the only sensor involved. [10,46]. Instead, similar to protein restriction, GCN2 may play a role in dampening growth signaling *via* the mechanistic target of rapamycin complex 1 pathway or in complimenting the action of other stress kinases such as AMP-activated protein kinase or the mitogen activated kinases MEK/ERK [47]. Irrespectively, it appears clear that the whole organism carries functional overlap in amino acid sensing and signaling in individual tissues. Such findings point to the necessity to further study these overlapping cellular responses to SAAR. 

## 4. Sulfur Amino Acid Restriction Promotes Gene-Specific Translation of ATF4 Regardless of eIF2 Phosphorylation

A key component of the canonical ISR response to deprivation of most amino acids is increased ATF4 synthesis which activates the expression of cytoprotective genes [36,48]. Rats fed an SAA-restricted diet for seven days showed higher levels of hepatic eIF2 phosphorylation and ATF4 protein expression [49,50]. In most studies utilizing SAA deprivation or prolonged SAAR, the phosphorylation of eIF2 corresponds with ATF4 synthesis but one should hesitate to conclude cause and effect. For example, it was found that hepatic *Atf4* expression was elevated in mice after two days of feeding an SAA restricted diet even though levels of eIF2 phosphorylation and GDP/GTP exchange rates on eIF2 were unchanged relative to animals fed a control diet. These results indicate an uncoupling between eIF2 phosphorylation and ATF4 synthesis in liver [44]. Interestingly, later timepoints in the same study showed the expected relationship between increased phosphorylation of eIF2, reduced rates of GDP/GTP exchange, and increased ATF4 target gene expression in liver. The early uncoupling between eIF2 phosphorylation and ATF4 target gene expression in response to SAAR suggests the presence of a non-canonical ISR; in other words, other post-transcriptional mechanisms may regulate ATF4 levels *in vivo*, such as altered protein stability. Additional research efforts are necessary to uncover the regulation of ATF4 by SAAR. 

## 5. Sulfur Amino Acid Restriction Promotes Expression of ATF4 Target Genes Which Includes Fibroblast Growth Factor 21

As detailed above, the ATF4-mediated transcriptional response is tailored to the specific cellular stress. Indeed, individual amino acid stress responses are heterogeneous and *in vitro* studies have revealed that the methionine-deprived transcriptional response is unique [23]. Furthermore, while both amino acid deprivation and restriction results in transcriptional changes in ATF4 target genes, the execution of the transcriptome favors apoptotic signaling only when the insufficiency is severely intense or sustained [36]. The timing and determinants of this shift in cell fate remains unclear and is likely a function of the interactions that occur between ATF4 and other bZIP transcription factors and the complex network of interacting binding proteins that act as transcriptional coactivators and corepressors. Intriguingly, much in this area remains to be uncovered. 

Among the ATF4 gene target identified under conditions of protein dilution and amino acid deprivation is fibroblast growth factor 21 (FGF21), an endocrine member of the FGF superfamily [51,52,53,54,55,56]. As a predominantly hepatic-derived endocrine signal of nutrient stress, FGF21 interacts with a number of target tissues [57]. Identification of FGF21 target tissues, including liver, various adipose deposits and regions of the brain have been made based on expression of the receptor, FGFR, and the requisite co-receptor β-klotho. Upon interaction with the receptor constellation, FGF21 results in stimulation of a number of metabolic effects, including increased mitochondrial uncoupling and thus energy expenditure as well as increased free fatty acid oxidation [57]. 

While various forms of amino acid insufficiency can induce the hepatic synthesis and secretion of FGF21 into the blood, the precise upstream mechanisms initially remained elusive. Involvement of the ISR in the physiological induction of FGF21 is supported by identification of up to three AAREs in the promoter region of *Fgf21* [58,59]. Corroborating these findings, mice respond to protein restriction by increasing ATF4 binding to AAREs in the *Fgf21* promoter region [60]. Accordingly, while the ATF4 binding coincided with increased circulating FGF21 in genetically intact animals, the response was perturbed in animals lacking *Gcn2*. Interestingly, loss of *Gcn2* did not completely abrogate the ATF4-mediated increase in FGF21, but rather caused a noticeable temporal delay. It was subsequently concluded that while GCN2/ATF4 signaling is imperative in the acute response to protein restriction, it is not essential; suggesting that upon loss of *Gcn2*, other regulators may facilitate ATF4-mediated induction of FGF21. Notably, this contrasts with drug-mediated amino acid starvation which requires GCN2 for hepatic expression of Fgf21 [54]. In total, these studies suggest that the canonical GCN2-eIF2-ATF4 axis contributes to but does not exclusively control *Fgf21* expression during amino acid insufficiency. 

The first evidence connecting FGF21 to the physiological response to SAAR came from a study showing that global loss of *Fgf21* prevents some of the metabolic improvements seen with SAAR [61]. Specifically, it was shown that male mice lacking *Fgf21* globally failed to increase energy expenditure as well as thermogenesis across adipose deposits. However, it was noted that mice lacking *Fgf21* did maintain hepatic lipogenic signaling—similar to the response in genetically intact animals. Furthermore, mice lacking *Fgf21* displayed normal hepatic ISR signaling, suggesting this signaling axis is dispensable.

In agreement with previous reports, it was found that *Gcn2* is dispensable in the SAAR-mediated induction of FGF21 and mice lacking *Gcn2* show delayed increases in energy expenditure [10]. Of note, it was observed that mice lacking *Gcn2* could sustain hepatic phosphorylation of eIF2 to SAAR, presumably by another eIF2 kinase, PERK. Based on these observations, it has been proposed that reduced ER abundance of a cysteine metabolite, glutathione (GSH), during SAAR might contribute to the activation of PERK (further detailed below) [10]. In addition, mice lacking *Gcn2* globally responded to acute SAAR by inducing hepatic *Fgf21* transcript abundancy without increased phosphorylation of eIF2 nor reduced activity of eIF2B in liver [44]. 

In summary, the currently available literature is supportive of FGF21 as a central mediator in the physiological response to SAAR. While the importance of FGF21 during SAAR is recognized, the upstream control remains elusive and appears complex in its nature, as pointed in a recent review [62]. In revealing the precise mechanisms behind the upstream control of FGF21 during SAAR, future efforts will need to consider the relationships between the ISR and other nutrient responsive signaling networks. 

## 6. Altered Feeding Behavior in Response to Sulfur Amino Acid Restriction: Implications for the ISR

Among the more intriguing phenotypical changes observed in SAA restricted rodents is the increase in relative food intake. This SAAR-mediated increase in food consumption is reported on extensively in rodents [4,16,63]. Notably, while the increased energy expenditure seen in SAA restricted animals has been well-delineated and found to be, at least in part, dependent on a number of mechanisms including β-adrenergic signaling, the mechanisms behind the hyperphagic response have been less clear, but have been reported to partly depend of β-adrenergic signaling [64,65]. 

In addition, FGF21 might, as discussed previously, play a role in altering the feeding behavior in response to SAAR [61]. Recent findings corroborate this notion, showing that injection of human recombinant FGF21 in male mice results in increased food intake simultaneous with reduced body weight [66]. Restriction of either leucine or methionine generates a similar increase in relative food intake; suggesting that the mechanism controlling hyperphagia is not unique to SAAR [24]. In light of this, it is important to draw attention to the numerous findings indicating the importance of the ISR and GCN2 in detecting amino acid devoid diets [67]. Specifically, leucine devoid diets, in contrast to leucine restriction, are initially avoided by genetically intact animals, leading to rapid food rejection and foraging. In contrast, mice lacking *Gcn2* in the anterior piriform cortex are unable to immediately sense and thus demonstrate delayed avoidance of the unbalanced diet [67]. Indeed, male mice with global loss of *Gcn2* do reduce food intake when chronically consuming a leucine devoid diet, highlighting the early role of *Gcn2* in instigating food aversion during exposure to amino acid devoid diets [46]. 

Collectively, while there is ongoing investigation into the specific temporal dependency of GCN2, the overall importance of the ISR in changing feeding behavior in response to amino acid insufficiency remains an interesting area of research warranting additional exploration [68,69].

## 7. Sulfur Amino Acid Restriction Results in Improved Oxidative Stress Defenses in Part *via* the Integrated Stress Response

Cysteine is a dispensable amino acid under conditions of adequate methionine because it can be synthesized from the methionine metabolite cystathionine, generated *via* the transsulfuration pathway. SAAR leads to observable changes in circulating levels of methionine metabolites. A series of publications shows that SAAR reduces levels of both total cysteine and cystathionine whereas total homocysteine levels increase [7,8]. Based on this, it is postulated that some of the effects seen in mice fed an SAA restricted diet are mediated by metabolites downstream of both methionine and cysteine. 

The importance of both methionine and cysteine metabolites is further emphasized when returning to some of the original work which shows that SAAR-mediated lifespan extension coincides with a robust increase in circulating levels of the cysteine metabolite GSH [70]. The major function of GSH is to act as an antioxidant in both tissue and in circulation, thus protecting cells from excess oxidative damage [71]. 

Studies note a negative correlation between advancing age and levels of circulating GSH [72]. In agreement, a reduction in circulating GSH with advancing age is observed in male rats fed a standard diet. However, rats fed an SAA restricted diet display maintained or elevated levels of circulating GSH, concomitant with a decrease in liver and kidney GSH, compared to control-fed rats [70]. Interestingly, these changes appear to have occurred as early as 8 weeks after initiation of SAAR and were maintained throughout the lifespan of the animals. Comparisons between different tissues show the reductions in GSH levels in liver and kidney are accompanied by a reduction in cysteine levels. Other organs did not differ in GSH or cysteine levels when compared to control-fed animals. Taken together, SAAR elicits metabolic adaptations that serve to optimize the synthesis of GSH and maintain elevated levels of the antioxidant in circulation, potentially at the expense of hepatic GSH levels. 

In support of the notion that altered levels of GSH are part of the physiological response to SAAR, additional studies confirm that mice subjected to an SAA restricted diet display reduced levels of the antioxidant in liver [10,44]. Concomitantly with reduced levels of hepatic GSH, increased phosphorylation of eIF2 is also observed, an effect that is sustained in mice lacking *Gcn2* and corresponds with phosphorylation of PERK. These findings were corroborated in a study showing that SAAR altered GSH tissue distribution and induced activation of PERK and phosphorylation of eIF2 in the liver without inducing ER stress-related gene expression [45]. 

It is hypothesized that PERK activation by SAAR increases activation of the bZIP transcription factor nuclear factor erythroid 2 related factor 2 (NRF2), with the purpose of increasing GSH production during both oxidative and ER stress [73]. Indeed, it is shown that SAAR increases signaling through NRF2, controlling transcriptional antioxidant response programs [10]. Collectively, these observations implicate the ISR in modulating the transsulfuration pathway and thus subsequently the methionine and cysteine metabolite GSH in the physiological response to SAAR.

Other studies postulate that the increased mitochondrial activity seen in various models of dietary restriction, including SAAR, is a function of increased ATF4 activity and might prompt increased antioxidant signaling *via* NRF2 binding to antioxidant response elements (AREs) [74,75]. Notably, the NRF2 gene itself contains at least two ARE-like elements in its proximal promoter region, of which at least one has been shown to bind NRF2 [76]. Furthermore, ATF4 is among the NRF2 dimerization partners and the genes regulated by NRF2 include those involved in GSH metabolism and antioxidant activity, linking ISR and SAAR to oxidative defenses [77]. 

Interestingly, earlier studies describe an increased cysteine requirement for mouse embryonic fibroblasts lacking *Atf4* to maintain homeostasis during GSH depletion, highlighting a role of the ISR in maintenance of adequate antioxidant defenses [78]. This observation was extended by a study showing that HepG2/C3A cells cultured in a cysteine depleted media display a significant reduction in GSH concentration [79]. In addition, the reduction in GSH concentration correlated with increased eIF2 phosphorylation. Additional microarray and gene expression data suggested that cysteine deprivation resulted in differential gene expression of AARE-containing genes. Taken together, accumulating literature supports the notion that the ISR plays a crucial role in regulating antioxidant defenses during SAAR.

Reviewing the annotated effects of SAAR on oxidative signaling leads one to suppose that these effects may contribute to other alterations in physiology. For example, rodents fed an SAA-restricted diet demonstrate increases in vascular endothelial growth factor (VEGF) *via* GCN2/ATF4-dependent signaling which in turn increases production of the proangiogenic gas, hydrogen sulfide (H_2_S) and promote skeletal muscle angiogenesis [80]. In this same report, SAA deprivation in human umbilical cord endothelial cells results in increased H_2_S production. Of interest, the production of H_2_S is mediated by the ATF4 target cystathionine-γ-lyase (CGL, encoded by *Cth*) and a recent report implicated the effects of H_2_S directly in the ISR. This study showed that the gas may cause a transient increase in eIF2 phosphorylation by inhibiting protein phosphatase 1c*via* persulfidation [81]. While these studies require further confirmation of the responsiveness *in vivo*, they support the importance of the ISR in the response to SAAR and highlight the wide spectrum of physiological and metabolic effects induced by the restriction of methionine and cysteine.

## 8. Perspectives

The ability of organisms to rapidly respond to changing environments, such as nutrient scarcity, is essential to survival and evolutionary fitness. However, recent decades have presented humans with a previously unencountered problem: chronic excess of dietary energy; leading to increased prevalence of metabolic ailments. Among the strategies employed to treat and prevent such metabolic ailments are various types of dietary restriction. The emergence of SAAR as a potential tool in improving metabolic health is intriguing, especially when considering the paradoxical nature of the response (i.e., eat more, weigh less, live longer). 

Based on the numerous promising findings in animal models, there is emerging interest in translating SAAR to humans, however there is only a limited number of published reports on the topic [82,83]. Nonetheless, since consumers of vegan and lacto-ovo-vegetarian diets generally have lower intake of SAAs, it has been proposed that one plausible strategy of implementing SAAR in humans is through such diets [84]. In agreement, a recent study reported that human omnivores subjected to a short-term vegan diet displayed increased levels of circulating FGF21, suggesting that such diets may indeed be translational alternatives to the SAAR utilized in rodent studies [15]. 

In reviewing the current rodent SAAR literature, it is apparent that much still remains to be understood. Among the questions requiring immediate attention is the apparent sex dimorphic response to SAAR. Two recent studies showed that both restriction and short-term deprivation of methionine results in a noticeable sex dimorphic response, with males generally presenting a more robust response compared to females [44,85]. Drawing parallels to recent caloric restriction studies, it appears that the sex dimorphic response to SAAR may recapitulate some of the differences between the biological sexes during caloric restriction [86]. Thus, by incorporating animals from both biological sexes in future studies, it will be possible to discern major sex differences that still remain largely unexplored. 

It will also be interesting to investigate the effects of the SAA restricted diet on reproduction. Indeed, studies in *Drosophila melanogaster* have shown that while dietary restriction, such as SAAR, results in extension of lifespan, it concomitantly decreases fecundity [87]. Future research might also focus on SAAR and reproductive success, including effects of in utero exposure to dietary ISR activation, much similar to the extensive literature surrounding fetal programming in response to in utero exposure to protein restriction [88,89,90].

Lastly, the involvement of the ISR in mediating lifespan extension in SAA restricted rodents needs additional investigation. With SAA restricted rodents living approximately 40% longer, there is an interest in understanding the underlying mechanisms and subsequently the translational aspects of this SAAR-mediated lifespan extension. To that end, it has recently been shown that progeroid mice fed an SAA-restricted diet display an extension of lifespan, indicative of possible therapeutic applications [91]. Collectively, this review summarizes the current understanding of how the evolutionary conserved ISR is involved in the physiological response to SAAR and highlights several areas that warrant additional research efforts.
